# Synthesis of Negatively Charged Polyol-Functional PSF Membranes with Good Hydrophilic and Efficient Boron Removal Properties

**DOI:** 10.3390/polym11050780

**Published:** 2019-05-01

**Authors:** Jinbo Jin, Xilan Du, Jie Yu, Shuhao Qin, Min He, Kaizhou Zhang, Jingkui Yang

**Affiliations:** 1 College of Materials and Metallurgy, Guizhou University, Guiyang 550025, China; jinjinbo66@163.com (J.J.); hemin851@163.com (M.H.); 2 National Engineering Research Center for Compounding and Modification of Polymeric Materials, Guiyang 550014, China; mydxl123@163.com (X.D.); Try-boat@163.com (K.Z.); jkyang2006@163.com (J.Y.)

**Keywords:** affinity surface, polyhydroxy, boron adsorption, superhydrophilic

## Abstract

Boron removal remains a major barrier to water purification, it is important to develop a specialized adsorption membrane for boron removal. By means of a simple and effective method, a hydrophilic membrane for boron removal with a polyhydroxy functional group on the surface was prepared. Firstly, a polysulfone (PSF) membrane was modified by co-depositing polyethyleneimine (PEI) with dopamine (DA) in one-step to produce amine-rich surfaces, then the DA/PEI-functionalized membranes were reacted with glycidol, with the prepared membranes corresponding to PSF-PDA/PEI membranes and PSF-diol membranes. The prepared membranes were characterized by water-uptake, FTIR, (X-ray photoelectron spectroscopy) XPS, (Field emission scanning electron microscope) FESEM, and zeta potential measurements. The hydrophilicity of the membrane was characterized by the static water contact angle (WCA) test. In addition, we systematically studied the impact of initial boron concentration, chelating time, and pH value on boron removal performance. The results showed that the PSF-diol membrane had strong hydrophilicity with a WCA of about 38°. The maximum adsorption capacity of boron appeared to be 1.61 mmol/g within 10 min at a boron concentration of 300 mg/L. Adsorption kinetics showed that saturation adsorption can be achieved in minutes at the initial concentration of 5 mg/L, which is beneficial to a rapid filtration process.

## 1. Introduction

Boron is one of the essential trace elements for plants and animals [1]. Appropriate boron can promote the transport of plant carbohydrates, regulate the synthesis of sugars, and promote cell division [2,3]. However, the difference between boron deficiency and boron poisoning is very small. When the concentration of boron in soil is above 2 mg/L, the plant will show boron poisoning, while when the boron content is lower than 1 mg/L, the plant will show boron deficiency [4,5]. For humans, excessive intake of boron can seriously affect people’s health, causing symptoms such as nausea, vomiting, headache, diarrhea, kidney damage, and even reproductive nervous system diseases [6]. Therefore, researchers have studied the problem of boron removal and proposed a variety of boron removal methods. So far, there are mainly adsorption method, precipitation method, liquid–liquid extraction method, electro-dialysis, ion-exchange resin method, electro-coagulation method, and membrane separation methods [7,8,9,10]. However, because of the low boron content, small radius, low electro-negativity, and other special properties, boron removal technology remains a technical problem [11,12].

Membrane separation technology is a new and efficient separation technology in water treatment, but it suffers from limitations such as the size of the separated substance and charge discrimination [13]. Affinity membrane separation is a new method with high selectivity and efficiency for the removal and enrichment of trace substances [14,15]. Therefore, combining the advantages of membrane technology and the characteristics of the boron element, it is necessary to develop a specialized adsorption membrane for boron removal. Polyhydroxy structure has been proved to have effective complexation with boron [16,17]. Shi et al. [15] blended a glycopolymer and polysulfone (PSF) for boron adsorption, and the results showed that boron uptake reached 0.2 mmol/g. By comparing the ligand structures of polyhydroxy polymers, Du et al. [18] found that the presence of the amino group and the branched chain structure are beneficial to improve the complexing efficiency. Recently, hyperbranched-polyol-tethered membranes were proved to be highly effective and fast adsorbent, but their preparation conditions are more demanding [19,20].

Dopamine (DA) is widely used to modify membrane materials due to its excellent auto-agglutination and hydrophilic properties [21,22]. The amino and carbonyl groups of polydopamine (PDA) can induce secondary modification reactions, which have attracted extensive attention [23,24]. The amino group of hyperbranched polyethyleneimine (PEI) can react with DA with Schiff base and Michael addition, so DA/PEI was deposited on the surface of a (Polyvinylidenefluoride) PVDF membrane surface; the layer can improve hydrophilicity and oil–water separation efficiency [25]. It was found that the PDA/PEI coating possesses a superhydrophilic membrane surface and the oil rejection is more than 98% [26].

**Figure 1:**
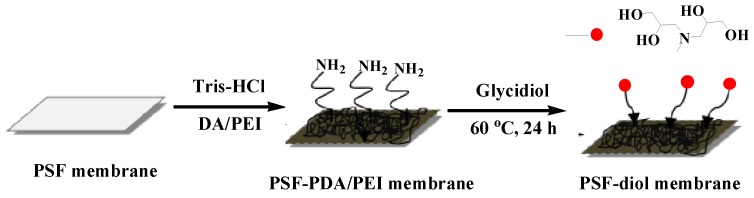
Schematic illustration for preparing polysulfone (PSF) composite membranes. DA—dopamine; PDA—polydopamine; PEI—polyethyleneimine.

Combined with the above experiments, we introduce a simple and highly efficient membrane for boron removal. Firstly, PSF membranes were modified by DA and PEI in one-step to produce amine-rich surfaces, then the DA/PEI-functionalized membranes were reacted with glycidol, so a composite membrane with polyhydroxy structure was obtained, with the prepared membranes corresponding to PSF-PDA/PEI membranes and PSF-diol membranes. The scheme is shown in Figure 1. The prepared membranes were confirmed by water-uptake measurements, FTIR, XPS, FESEM, and zeta potential tests. The hydrophilicity of the membrane was characterized by the static water contact angle (WCA) test. In addition, we systematically studied the impact of initial boron concentration, chelating time, and pH value on boron removal performance. We also used Freundlich and Langmuir isothermal adsorption models to fit the boron adsorption properties of thermodynamics.

## 2. Experiments

### 2.1. Materials

Polysulfone (PSF, M_n_ = 58,000 g/mol, BASF, Shanghai, China) and PEI (M_w_ = 6500 g/L) were supplied by Sigma-Aldrich (Shanghai, China). Glycidol (97%), dopamine hydrochloride (DA-HCl) (98%), and tris(hydroxymethyl) aminomethane (Tris) were obtained from J&K (Beijing, China). Polyvinylpyrrolidone (PVP) and N-methylpyrrolidone (NMP) were purchased from Sinopharm Group Chemical Reagents Co., Ltd. (Beijing, China). All other chemicals were analytical grade and were used without further purification.

### 2.2. Procedure of PSF-PDA/PEI Membrane Preparation

In this paper, a PSF membrane was prepared following the steps in reference [18] with a mass ratio of PSF/PVP/NMP = 18/75/7. The PSF-PDA/PEI membrane was prepared in the following steps. Firstly, the PSF membrane was washed clean and immersed in Tris buffer solutions (15 mM, pH = 8). Then DA and PEI were added into the buffer solutions with different mass ratios. The reaction was carried out at 25 °C for 10 h. After reaction, the prepared membrane was cleaned with deionized water to remove adsorbed molecules.

### 2.3. Procedure of PSF-Diol Membrane Preparation

The PSF-diol membrane was prepared by the reaction of amino groups on the PSF-PDA/PEI membrane with glycidol. At first, the PSF-PDA/PEI membrane was immersed in 100 mL of ethanol solution in 500 mL three-necked flasks, then glycidol was added dropwise to the solution at a 60 °C reflux reaction for 24 h with magnetic stirring [27]. After that, the membrane was washed thoroughly with ethanol and ultrapure water before testing.

### 2.4. Membrane Characterization

The surface chemical compositions of the original and modified PSF membranes were analyzed by XPS (Thermo Fisher, K-alpha, Shanghai, China) and FTIR (Bruker, Vector-22, Beijing, China) measurements. The surface and cross morphology of the membrane was characterized by FESEM (Quanta FEG250, Beijing, China) tests. A zeta potential analyzer was used to characterize the surface charge characteristics of the membranes (Shanghai, China). The static water contact angles (WCA) of PSF, PSF-PDA/PEI, and PSF-diol membranes were calculated by a Kruss instrument (Hamburg, Germany).

### 2.5. Water-Uptake Test

The water-uptake test was used to character the water absorption mass of the PSF-PDA/PEI membrane after immersion in deionized water for 24 h. The water uptake can be calculated by the equation:

(1) $$W_{\mathrm{up}}=\frac{M_{\mathrm{wet}}-M_{\mathrm{dry}}}{M_{\mathrm{dry}}}\times 100\%,$$

where *W*_up_ stands for the water uptake (%), *M*_dry_ and *M*_wet_ represent the mass of the membrane before and after water absorption, respectively.

### 2.6. The Water Flux Test

The water fluxes of the original membrane and the modified membranes were measured with a cross-flow device at 0.1 MPa, 25 °C. The membrane was cleaned and pressurized before testing. The water flux was measured by the following equation:

(2) $$F=\frac{V}{S\cdot t},$$

where *F* represents the water flux (L/m^2^h), *V* is the volume (L) of water through the device in a given period of time (h), *S* is the effective area (cm^2^) of the membrane, and *t* is the penetration time (h).

### 2.7. Boron Adsorption Experiments

A certain mass of PSF-diol membrane was cleaned with methanol, and then the membrane was put into a solution with a known boron concentration, followed by a shock adsorption reaction for 3 h. Finally, the boron content was tested by the curcumin method [18]. The quantitative value of boron adsorbed is calculated using the following equation:

(3) $$q=\frac{(C_{0}-C_{\mathrm{eq}})V}{mM},$$

where *C*_0_ is the initial boron concentration (mg/L), and *C*_eq_ represents the boron concentration at the adsorption equilibrium (mg/L). *V* could be the volume of the solution (L) and *m* is the mass of the membrane in the dry state (g). *M* is the molecular weight of the boric acid (g/mol).

## 3. Results and Discussion

### 3.1. Water Uptake of Membranes

**Figure 2:**
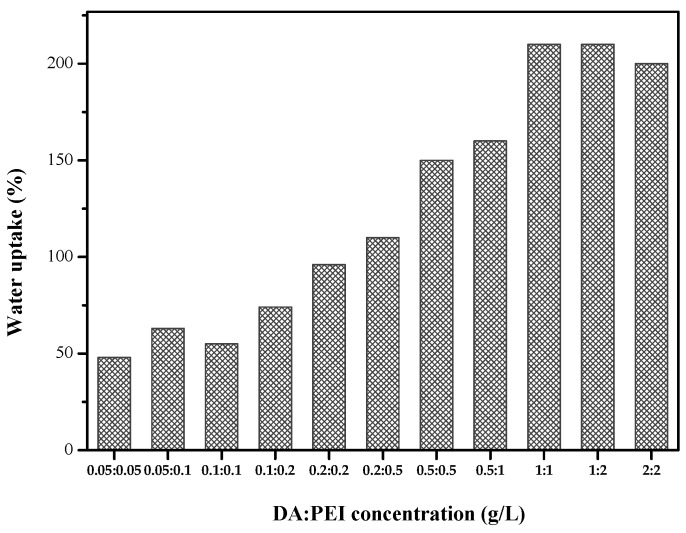
Water uptake of the DA/PEI-modified PSF membranes.

We can see from Figure 2 that when the concentration of DA and PEI is 1 g/L, the water uptake reaches the optimal level. When the concentration increased, the water absorption rate changes little. High water uptake represents high hydrophilic, therefore, when the concentration of DA/PEI is 1:1 g/L, the prepared membrane has good hydrophilicity properties.

**Figure 3:**
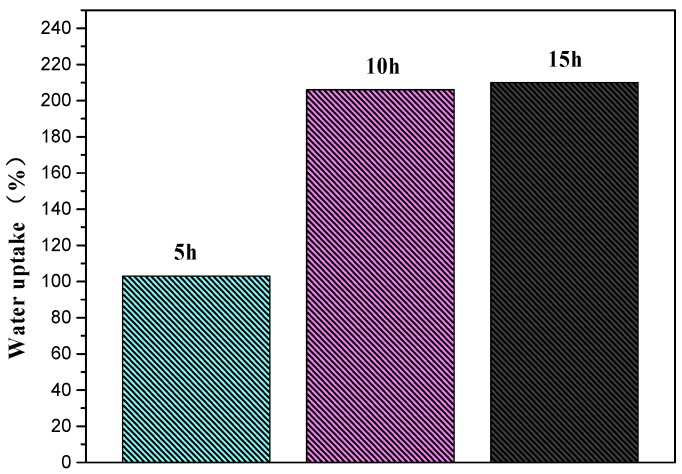
Water uptake of PSF-PDA/PEI with selected DA/PEI concentration (1:1 g/L) membranes at different deposition times.

Under the condition of DA/PEI concentration of 1:1 g/L, we went on to investigate the influence of deposition time on the water-uptake performance of the PSF-PDA/PEI membranes. As can be seen from Figure 3, when the deposition time was 10 h, the water uptake of composite membranes reached saturation. With the extension of deposition time, the water uptake did not increase significantly. To get the optimum test condition, we selected the deposition time as 10 h and the concentration ratio of DA/PEI is 1:1 g/L as a second reaction.

### 3.2. Membrane Surface Structure Characterization

#### 3.2.1. Surface Chemical Composition

**Figure 4:**
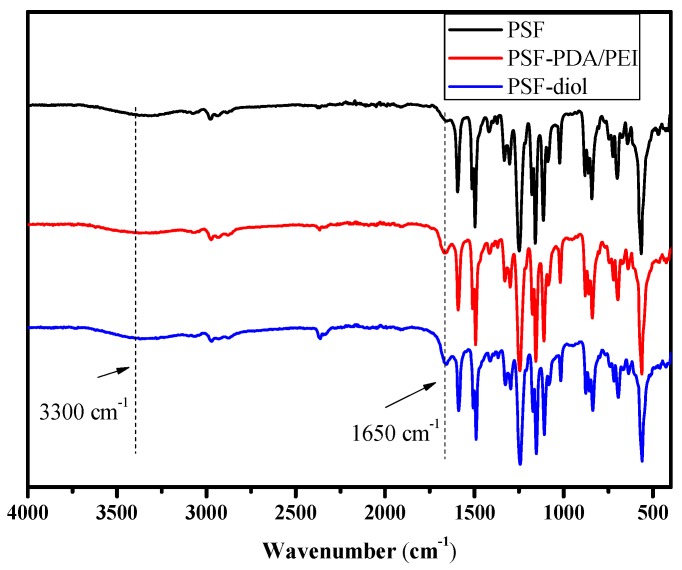
ATR-FTIR spectra of the PSF, PSF-PDA/PEI, and PSF-diol membranes.

The FTIR spectra of PSF, PSF-PDA/PEI, and PSF-diol membranes are shown in Figure 4. Compared with the original film, the vibration peak of the C=C double bond on the benzene ring at 1650 cm^−1^ of the composite membrane was enhanced, which was caused by the introduction of dopamine and polyethyleneimine on the surface of the composite membrane. In addition, the stretching vibration peaks of O–H and N–H appeared at 3300 cm^−1^ of the composite membrane, which also proved the successful preparation of the composite membrane [27,28]. Due to the residual PVP in the original PSF membrane, a weak carbonyl absorption peak appears. However, the peak intensity of C=O was significantly increased after the co-deposition of DA/PEI. All these phenomena indicate that dopamine and polyethyleneimine successfully deposited on the surface of the polysulfone membrane.

**Figure 5:**
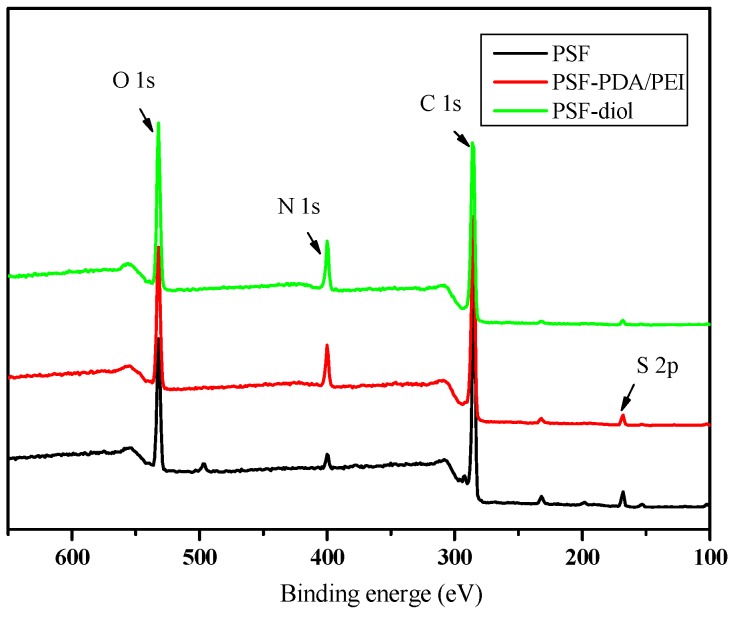
XPS spectra of PSF, PSF-PDA/PEI, and PSF-diol membranes.

We further characterized the surface element content of the base and composite membranes by XPS analysis. Figure 5 shows the XPS spectra of the original and composite membranes. As shown in Figure 5, a C1s emission peak appeared in the primary membrane at 285.1 eV. Due to the presence of the sulfone group in the primary membrane, strong emission peaks belonging to S2p and O1s appeared at 168.6 and 532.1 eV, respectively. Due to the presence of PVP residue in the original membrane, N1s peak occurs at 399.8 eV, which is consistent with the result of the FTIR test. On the PSF-PDA/PEI membrane, the contents of O and N increased obviously. In addition, after reaction with epoxide, the O content increased on the membrane surface further. Table 1 shows elemental surface composition of original and modified membranes. We can see that the atomic ratio of O/N in PSF-diol membrane is higher than that in the PSF-PDA/PEI membrane, which is consistent with the molecular structure of the membrane surface. This structure further proves that the composite membrane with a polyhydroxy structure on the surface was obtained by the co-deposition experiment.

#### 3.2.2. Surface and Cross-Sectional Morphologies of Membranes

**Figure 6:**
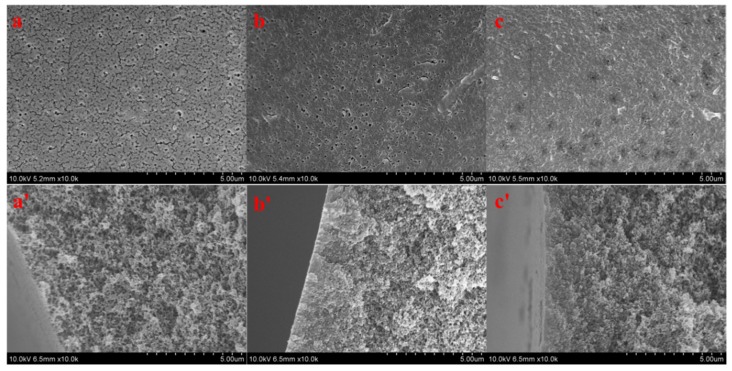
Surface and cross-sectional morphologies of PSF (**a**,**a′**), PSF-PDA/PEI (**b**,**b′**), and PSF-diol (**c**,**c′**) membranes.

Figure 6 shows the surface and cross-sectional morphologies of PSF (Figure 6a,a′), PSF-PDA/PEI (Figure 6b,b′), and PSF-diol (Figure 6c,c′) membranes. We can see their differences clearly from the outside. After the co-deposition of DA and PEI, the membrane pores on the surface of the composite membrane are smaller than those of the original film, and the outer surfaces of PSF-diol (Figure 6c) became denser than PSF (Figure 6a) and PSF-PDA/PEI (Figure 6b). With the introduction of DA and PEI on the membrane surface and the preparation of the composite membrane with a polyhydroxy structure, the hydrophilicity of the composite membrane was significantly improved, but at the same time, the presence of the coating reduced the membrane pores. In addition, no significant changes were observed from the membrane’s section structure, indicating that the reaction mostly occurred on the membrane surface and had little effect on the section.

#### 3.2.3. Zeta Potential Tests

**Figure 7:**
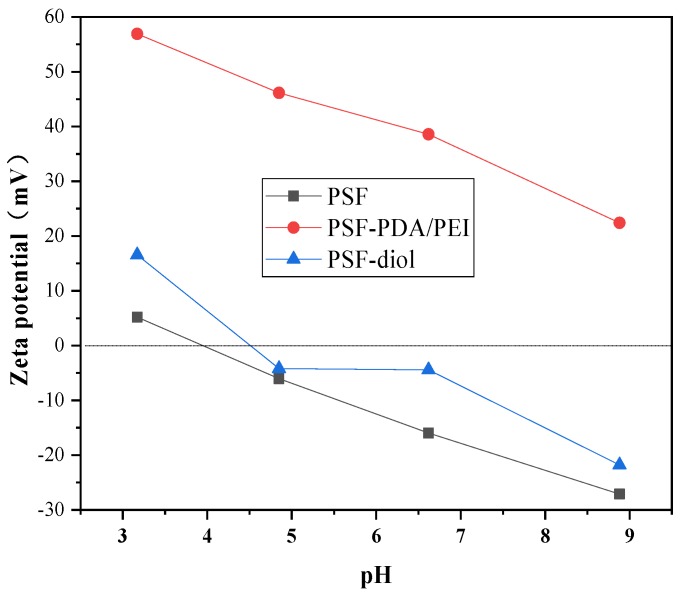
The zeta potential for PSF-PDA/PEI and PSF-diol membranes in different pH.

Membrane zeta potential is used to characterize the amount of the charge density on the membrane surface [6]. Figure 7 shows the zeta potential of PSF-PDA/PEI and PSF-diol membranes within the pH range of 3–9. We can clearly observe that the PSF-PDA/PEI membrane has a significant positive charge, which is caused by the amino group on the surface. Shi et al. [28] pointed out that a PEI coating can obviously increase the positive charge of the membrane surfaces, which was due to the hydrolysis reaction of excess amine groups in PEI. After the reaction with glycidol, the potential PSF-diol is determined by the quaternary ammonium cations and hydroxide ion. The surface of the PSF-diol membrane is negatively charged due to the presence of a great quantity of hydroxyl groups [3].

#### 3.2.4. WCA Measurements

**Figure 8:**
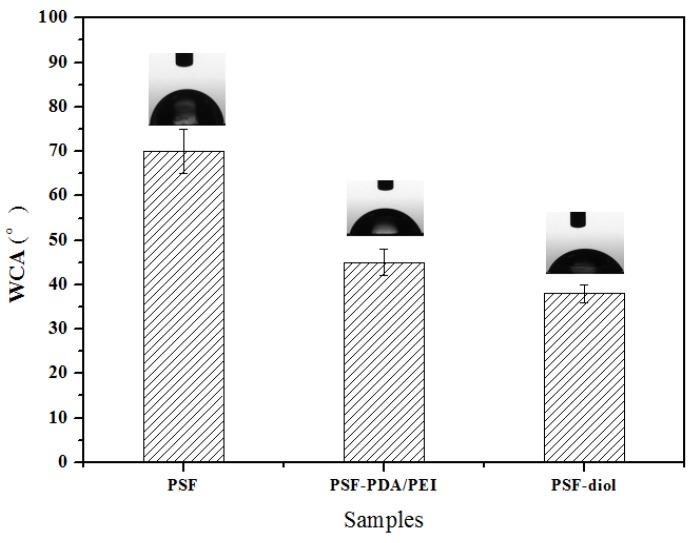
Water contact angle (WCA) of PSF, PSF-PDA/PEI, and PSF-diol membranes.

In this paper, the static water contact angle test was used to determine the relative hydrophilicity of the membrane surface at least five locations. As seen in Figure 8, the WCA of origin membrane was 70° ± 5°, after coating by DA and PEI, the WCA of the PSF-PDA/PEI membrane reduced to about 45° ± 3°. The PSF-diol membrane had strong hydrophilicity and the WCA was an average of 38°. The results indicated that the hydrophilicity of the membrane surface was improved obviously after the introduction of hydrophilic functional groups such as amino and hydroxyl groups. The hydrophilicity of the membrane surface is related not only to the hydrophilic and hydrophobic properties of functional groups contained on the membrane surface but also to the roughness and electric charge of the membrane surface [29].

### 3.3. Membrane Flux Performance

**Figure 9:**
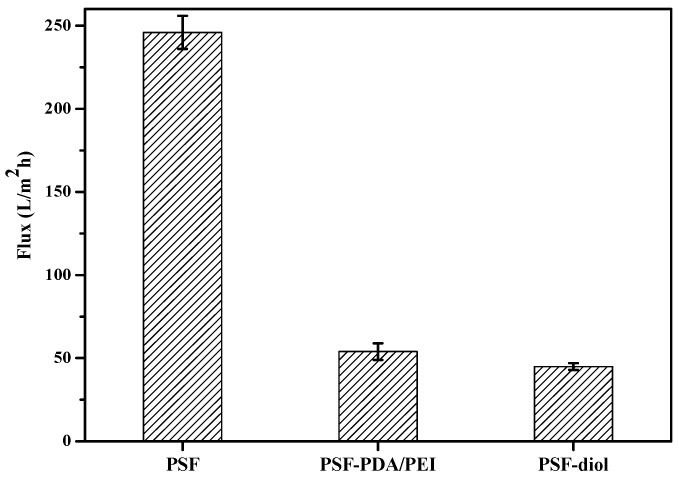
The flux of PSF, PSF-PDA/PEI, and PSF-diol membranes.

The pure water fluxes of the original and modified membranes were measured at 0.1 MPa, 25 °C. As can be seen from Figure 9, the original PSF membrane flux was 246 L/m^2^ h, while the water fluxes of PSF-PDA/PEI and PSF-diol membranes were 54 and 45 L/m^2^ h, respectively. Surface hydrophilicity is one of the main factors affecting water flux. The decrease of the water flux of the composite membrane was caused by the blockage of the membrane holes by the composite layer after the membrane surface was coated. Zin et al. [26] confirmed that the reaction between DA and PEI enhances the stability of the coating of membrane surface, which indicates that the coating of PDA/PEI on the surface of PSF membrane was successful.

### 3.4. Boron Removal Experiments

**Figure 10:**
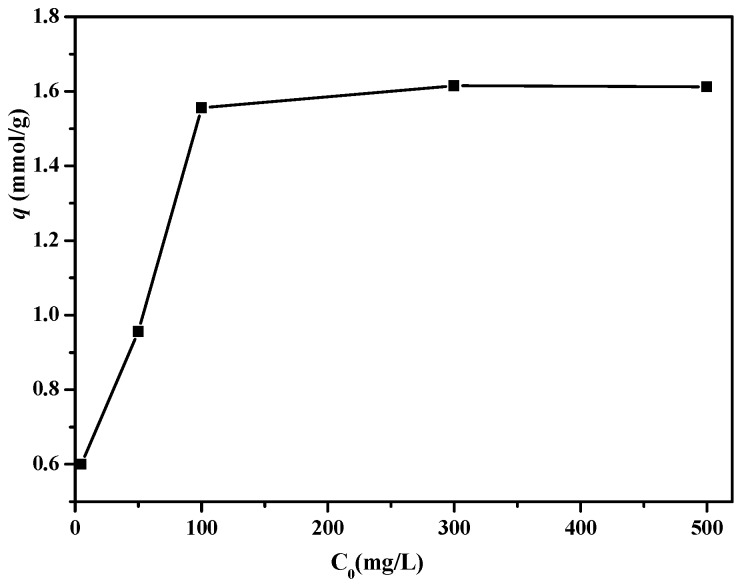
Effect of the initial boron concentration on boron uptake of PSF-diol membrane (pH = 8, T = 25 °C).

The influence of initial boron concentration on the adsorption performance of PSF-diol membrane is shown in Figure 10. It can be seen from the figure that the adsorption amount of boric acid by PSF-diol membrane increases with the increase of initial boron concentration. When the initial concentration was 300 mg/L, the maximum adsorption capacity appeared to be 1.61 mmol/g, and then the adsorption platform appeared, this is because the surface of the PSF-diol membrane has a large number of ortho-hydroxyl groups that can be complexed with boric acid.

In order to further understand the mechanism of boron adsorption, Langmuir and Freundlich isothermal adsorption models were used to fit the experimental data. The Langmuir isotherm model assumes a single-layer adsorption on a homogeneous surface. The Freundlich isothermal model assumes multilayer heterogeneous adsorption at different energy adsorption sites. Their equations are as follows:

(4) $$\frac{C_{\mathrm{eq}}}{q}=\frac{C_{\mathrm{eq}}}{q_{\max}}+\frac{1}{bq_{\max}},$$

(5) $$q=K_{f}C_{\mathrm{eq}}^{1/n},$$

where, *q*_max_ (mmol/g) and *C*_eq_ (mg/L) are the theoretical maximum adsorption capacity and adsorption concentration of the affinity membrane in the saturated state, respectively; *b* (L/mg) is the adsorption coefficient, and *K_f_* and 1/*n* are Freundlich constants (mmol/g).

**Figure 11:**
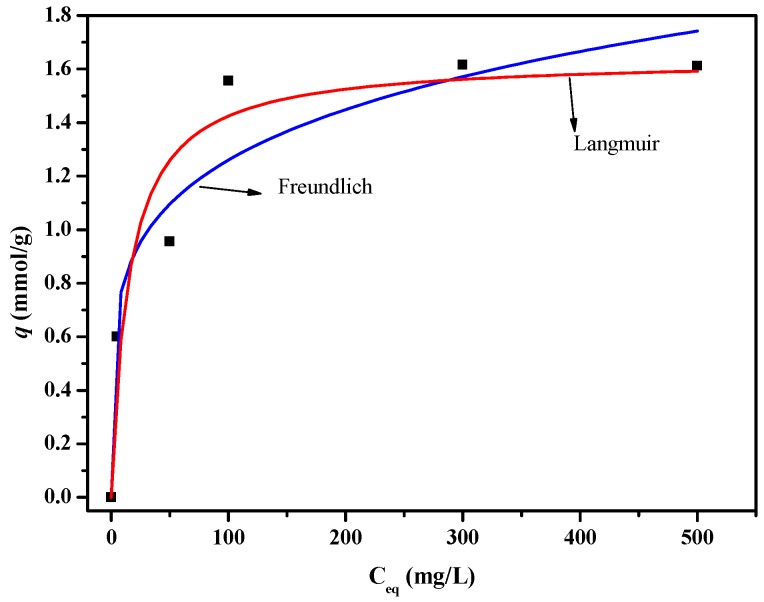
Boron adsorption isotherms of PSF-diol membrane.

Figure 11 shows the adsorption isotherm curves of the PSF-diol membrane, and the isotherm parameters are listed in Table 2. We can see from the results that compared with the Freundlich model, the R^2^ value fitted by Langmuir is the highest, and the calculated value of *q*_max_ is close to the experimental value, indicating that the adsorption of boric acid on the membrane surface is uniform monolayer adsorption.

**Figure 12:**
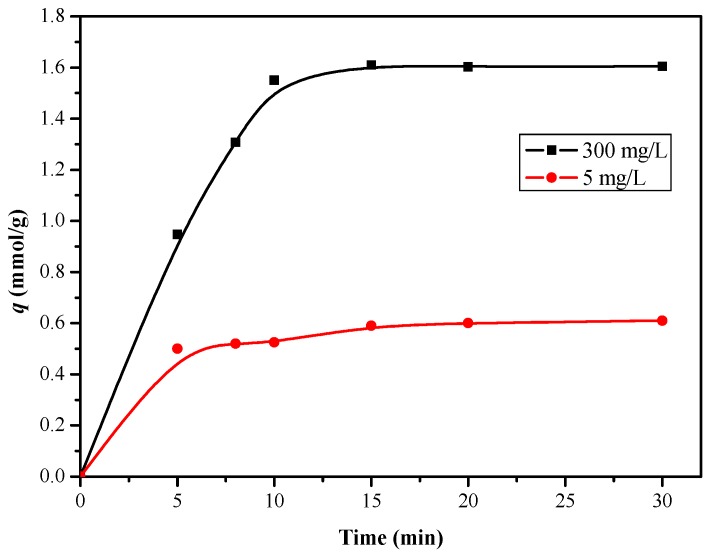
Effect of time on boron uptake of PSF-diol membrane (pH = 8.0, T = 25 °C).

As can be seen from Figure 12, the adsorption amount of boron increased sharply at the beginning, and then reached adsorption saturation at 10 min. The results showed that the polyhydroxy functional groups on the affinity membrane surface can achieve a high efficiency of boron adsorption in a short time. As it is known, the adsorption kinetics of boric acid on the affinity membrane is mainly affected by boron diffusion between layers of the polyhydroxy copolymer on the surface of the affinity film and the interaction between boric acid and hydroxyl functional groups. Meng et al. [19] pointed out that the hyperbranched polymer scaffold is beneficial to superior boron-uptake performance.

**Figure 13:**
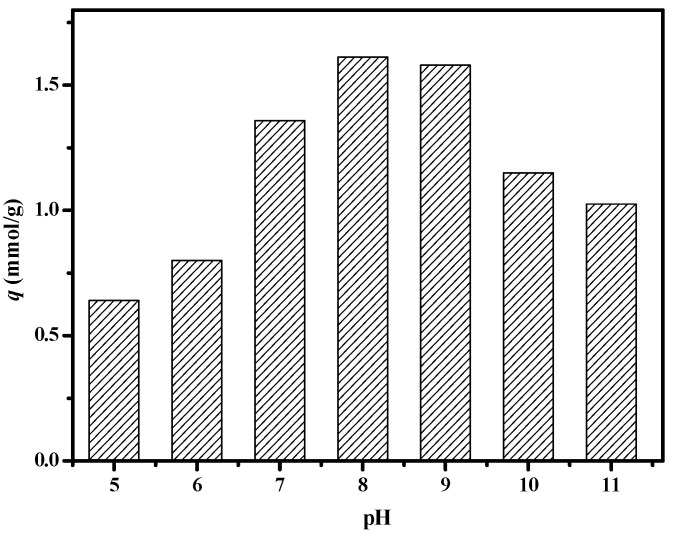
Effect of pH value on boron uptake of PSF-diol membrane (*C*_0_ = 300 mg/L, T = 25 °C).

We investigated the effect of pH values on boron adsorption. It can be seen from Figure 13 that the composite membrane has a good adsorption effect on boric acid under weakly alkaline conditions. When the pH is 8.0, the adsorption capacity reaches the maximum value. This is because under acidic conditions, there is a large amount of hydrogen ions in the solution, which will inevitably lead to its complexation reaction moving toward dissociation, and the adsorption capacity of boric acid decreases. Under alkaline conditions also, the adsorption capacity decreased because of the adsorption competition between borate ions and a large number of hydroxyl groups in the system.

**Figure 14:**
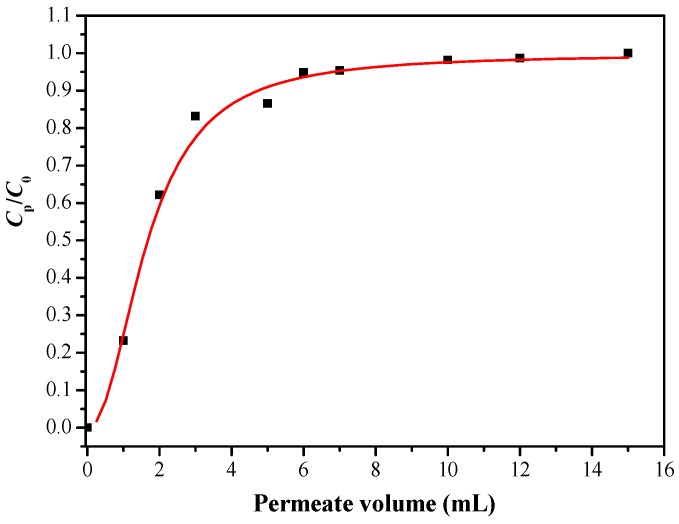
Breakthrough curve of PSF-diol membrane (*C*_0_ = 5 mg/L, pH = 8).

Boron rejection percentage is an important parameter for evaluating adsorption performance in actual operation. Considering the actual content of boron in seawater, we chose the initial boron concentration of 5 mg/L. The dynamic boron adsorption experiment of the modified membrane was studied by using a dead-end filter model. The device was connected to a peristaltic pump to control a constant flow rate of 0.5 mL/min, and the concentration of the permeable solution (*C*_p_) was measured at certain volume intervals. The boron adsorption penetration curve is shown in Figure 14. It can be seen that the ratio of *C*_p_/*C*_0_ changed rapidly at the beginning, when the volume of the filtrate reached 1 mL, the ratio of *C*_p_/*C*_0_ was 0.23, which means the boron rejection percentage was 77%. When the volume of the filtrate reached 6 mL, the boron rejection performance did not fluctuate significantly, indicating a saturation adsorption, which is consistent with the results of the static boron adsorption experiment.

## 4. Conclusions

Through the co-deposition of DA and PEI on the surface of a PSF membrane, amine-rich surfaces were produced, then the DA/PEI-functionalized membranes were reacted with glycidol. The modified membranes of PSF-PDA/PEI and PSF-diol showed more hydrophilicity. Because the membrane surface was coated by a layer of polymer, the water flux of the prepared membrane decreased. The adsorption experiments on boric acid show that the PSF-diol membrane has high efficiency and fast adsorption of boron. The maximum adsorption capacity of boron appeared to be 1.61 mmol/g within 10 min at an initial boron content of 300 mg/L. The Langmuir and Freundlich isothermal adsorption models were used to fit the experimental data. The results showed that the fitting value of the Langmuir model was closer to the experimental value, indicating that the adsorption process of boric acid on the membrane was homogeneous single-layer adsorption. Adsorption kinetics showed that saturation adsorption can be achieved in minutes at the initial content of 5 mg/L, which is beneficial to a rapid filtration process. The composite membrane prepared in this paper has certain potential value in the field of boron removal in water treatment. However, the permeability of the composite membrane materials needs to be further improved. In the next step, we will improve the permeability of the composite membrane on the premise of ensuring the effect of boron removal.

## Figures and Tables

**Table 1:** Elemental surface composition of original and modified membranes.

Membranes	Atomic Percent (mol %)				Atomic Ratio
	C	O	N	S	O/N
PSF	72.61	18.45	3.51	2.7	5.26
PSF-PDA/PEI	70.97	19.55	7.83	1.64	2.50
PSF-diol	67.07	23.45	8.47	1	2.77

**Table 2:** List of isotherm parameters for boron adsorption (pH = 8.0, T = 25 °C).

Membrane	Langmuir Constants			Freundlich Constants		
	*q_max_* (mmol/g)	*b* (L/mmol)	R^2^	*K_f_* (mmol/g)	1/*n*	R^2^
PSF-diol	1.64	0.07	0.9322	0.49	0.20	0.8452
